# Plasma biomarkers of neurodegeneration in patients and high risk subjects with Lewy body disease

**DOI:** 10.1038/s41531-024-00745-8

**Published:** 2024-07-31

**Authors:** Keita Hiraga, Makoto Hattori, Yuki Satake, Daigo Tamakoshi, Taiki Fukushima, Takashi Uematsu, Takashi Tsuboi, Maki Sato, Katsunori Yokoi, Keisuke Suzuki, Yutaka Arahata, Yukihiko Washimi, Akihiro Hori, Masayuki Yamamoto, Hideaki Shimizu, Masakazu Wakai, Harutsugu Tatebe, Takahiko Tokuda, Akinori Nakamura, Shumpei Niida, Masahisa Katsuno

**Affiliations:** 1 Department of Neurology, Nagoya University Graduate School of Medicine, Nagoya, Japan; 2 Department of Neurology, Daido Hospital, Nagoya, Japan; 3 Department of Neurology, National Hospital for Geriatric Medicine, National Center for Geriatrics and Gerontology, Obu, Aichi Japan; 4 Innovation Center for Translational Research, National Center for Geriatrics and Gerontology, Obu, Aichi Japan; 5 Department of Comprehensive Care and Research on Memory Disorders, National Center for Geriatrics and Gerontology, Obu, Aichi Japan; 6 Kumiai Kosei Hospital, Takayama, Gifu Japan; 7 Medical Examination Center, Daido Clinic, Nagoya, Japan; 8 Chutoen General Medical Center, Kakegawa, Shizuoka Japan; 9 Department of Functional Brain Imaging, Institute for Quantum Medical Science, National Institutes for Quantum Science and Technology, Chiba, Japan; 10 Department of Biomarker Research, Center for Development of Advanced Medicine for Dementia, National Center for Geriatrics and Gerontology, Obu, Aichi Japan; 11 Core Facility Administration, Research Institute, National Center for Geriatrics and Gerontology, Obu, Aichi Japan; 12 Department of Clinical Research Education, Nagoya University Graduate School of Medicine, Nagoya, Japan

**Keywords:** Parkinson's disease, Dementia, Prognostic markers

## Abstract

Comorbid Alzheimer’s disease (AD) neuropathology is common in Lewy body disease (LBD); however, AD comorbidity in the prodromal phase of LBD remains unclear. This study investigated AD comorbidity in the prodromal and symptomatic phases of LBD by analyzing plasma biomarkers in patients with Parkinson’s disease (PD) and dementia with Lewy bodies (DLB) and individuals at risk of LBD (NaT-PROBE cohort). Patients with PD (PD group, *n* = 84) and DLB (DLB group, *n* = 16) and individuals with LBD with ≥ 2 (high-risk group, *n* = 82) and without (low-risk group, *n* = 37) prodromal symptoms were enrolled. Plasma amyloid-beta (Aβ) composite was measured using immunoprecipitation-mass spectrometry assays. Plasma phosphorylated tau 181 (p-tau181), neurofilament light chain (NfL), and alpha-synuclein (aSyn) were measured using a single-molecule array. Plasma p-tau181 levels were higher in the PD and DLB groups than in the low-risk group. Aβ composite level was higher in the DLB group than in the high-risk group. AD-related biomarker levels were not elevated in the high-risk group. NfL levels were higher in the high-risk, PD, and DLB groups than in the low-risk group. In the PD group, Aβ composite was associated with cognitive function, p-tau181 with motor function and non-motor symptoms, and NfL with cognitive and motor functions and non-motor symptoms. In the high-risk group, NfL was associated with metaiodobenzylguanidine scintigraphy abnormalities. The PD and DLB groups exhibited comorbid AD neuropathology, though not in the prodromal phase. Elevated plasma NfL levels, even without elevated AD-related plasma biomarker levels, may indicate aSyn-induced neurodegeneration in the LBD prodromal phase.

## Introduction

Lewy body disease (LBD) includes Parkinson’s disease (PD) and dementia with Lewy bodies (DLB), which are neurodegenerative disorders associated with intra-neuronal alpha-synuclein (aSyn) accumulation. Prodromal symptoms of LBD, including dysautonomia, hyposmia, and rapid eye movement sleep behavior disorder (RBD), precede the onset of motor or cognitive dysfunction by 10–20 years and are considered essential for the pre-onset risk assessment of LBD development^1^.

In our previous high-risk cohort study for LBD, we found that 5.7% of healthy participants aged ≥50 years had ≥2 prodromal symptoms; we defined these participants as high-risk individuals^2^. These participants had mild cognitive decline and hyposmia compared with low-risk participants with no prodromal symptoms. Approximately one-third of the high-risk individuals had defects in dopamine transporter (DaT) single-photon-emission computed tomography (SPECT) or cardiac metaiodobenzylguanidine (MIBG) scintigraphy, and the prevalence of abnormalities on DaT-SPECT was 4 times higher in the high-risk individuals than that in the low-risk individuals^3^.

In PD and DLB, limbic and neocortical aSyn pathology is associated with dementia; furthermore, previous postmortem brain studies demonstrated that comorbid Alzheimer’s disease (AD) neuropathology is associated with the progression of cognitive impairment. More than 70% of patients with DLB and approximately 50% of patients with PD dementia (PDD) have comorbid AD neuropathology^4,5^. Understanding the temporal progression of comorbid AD neuropathology is crucial for comprehending the motor and cognitive trajectories in LBD, and AD-related molecules may serve as a potential therapeutic target. Recently, AD-related plasma biomarkers, such as amyloid-beta (Aβ) composite (combination biomarker of amyloid-beta precursor protein (APP)_669-711_/Aβ_1-42_ and Aβ_1-40_/Aβ_1-42_ ratios) and phosphorylated tau 181 (p-tau181), have garnered attention^6,7^. In addition, plasma neurofilament light chain (NfL) is regarded as a reliable biomarker for various neurodegenerative diseases^8^. Although recent studies have examined AD-related plasma biomarkers in patients with PD and DLB^9,10^ and plasma NfL in patients with idiopathic RBD^11,12^, there is a lack of detailed studies on AD comorbidity in the prodromal phase of LBD.

This study measured and analyzed four plasma biomarkers, Aβ composite, p-tau181, NfL, and aSyn, in high-risk and low-risk individuals, participating in the NaT-PROBE study, as well as in patients with PD and DLB.

## Results

### Participant characteristics

There were more male participants in the low- and high-risk groups than in the PD and DLB groups. The PD and DLB group participants were significantly older than the low- and high-risk group participants. Among the high-risk participants, 36.6% had abnormalities in either DaT or MIBG, consistent with the findings in our previous study^3^. All patients with PD and DLB who underwent DaT or MIBG before the study inclusion exhibited these abnormalities. The PD and DLB group participants, though not the high-risk group participants, had worse MoCA-J scores compared with the low-risk group participants. Two patients with PD and three with DLB could not complete the Stroop test, and one patient with DLB could not complete the line orientation test. The average Hoehn and Yahr Scale score was similar between the PD and DLB groups. The PD and DLB group participants, though not those in the high-risk group, had worse MDS-UPDRS III scores. The high-risk participants who were selected based on the SCOPA-AUT, SAOQ, and RBDSQ scores had worse BDI-II, ESS, PDQ-39, and QUIP scores than the low-risk participants. The PD and DLB group participants had worse scores on these questionnaires as well, except for QUIP (Table 1).

**Table 1:** Background characteristics of the participants

	Low-risk (LR)	High-risk (HR)	PD	DLB	*p* value		
					LR vs HR	LR vs PD	LR vs DLB
Number (M:F)	37 (26:11)	82 (65:35)	84 (44:40)	16 (8:8)	0.822^a^	0.295	0.411
Age, years	63.8 (5.2)	64.9 (7.6)	68.8 (9.4)	78.4 (5.4)	0.909^b^	0.008	<0.001
Education, years	14.2 (1.9)	13.5 (2.1)	13.3 (3.0)	12.4 (3.9)	0.530^b^	0.246	0.110
DaT abnormal, %	3/37 (8.1)	21/82 (25.6)	43/43 (100)	12/12 (100)	0.028^c^		
MIBG abnormal, %	3/37 (8.1)	15/82 (18.3)	41/48 (85.4)	7/9 (77.8)	0.178^c^		
DaT and/or MIBG abnormal, %	3/37 (8.1)	30/82 (36.6)	62/62 (100)	15/15 (100)	<0.001^c^		
Disease duration, years	NA	NA	5.9 (4.9)	3.7 (3.9)			
MoCA-J	27.1 (2.4)	26.7 (2.9)	24.5 (4.0)	14.7 (6.9)	0.782^d^	0.048	<0.001
Stroop test part 2 - part 1, sec	9.7 (4.9)	12.7 (8.6)	21.6 (33.7)^e^	46.11 (34.70)^e^	0.597^d^	0.140	0.004
Line orientation test	18.2 (2.3)	17.0 (2.9)	15.8 (2.9)	12.9 (3.8)^f^	0.100^d^	0.008	<0.001
Hoehn and Yahr	0.0 (0.0)	0.0 (0.0)	2.1 (0.9)	2.8 (1.4)			
LEDD	0.0 (0.0)	0.0 (0.0)	403.1 (388.3)	25.0 (77.5)			
MDS-UPDRS III	2.2 (2.5)	4.4 (4.1)	25.0 (10.3)	28.9 (22.1)	0.488^d^	<0.001	<0.001
Rigidity	0.2 (0.6)	0.4 (0.7)	3.6 (3.1)	2.6 (2.6)	0.721^d^	<0.001	0.038
Tremor	0.4 (0.9)	0.5 (0.9)	3.8 (4.2)	2.0 (5.5)	0.969^d^	<0.001	0.886
Bradykinesia	1.4 (1.7)	2.3 (2.4)	12.5 (5.6)	16.3 (10.8)	0.296^d^	<0.001	<0.001
Axial signs	0.2 (0.6)	1.2 (1.4)	5.2 (3.8)	8.1 (6.2)	0.126^d^	<0.001	<0.001
SCOPA-AUT	1.9 (1.7)	10.2 (5.1)	11.6 (8.4)	13.4 (8.8)	<0.001^d^	<0.001	<0.001
SAOQ, %	99.7 (1.1)	83.2 (25.9)	67.9 (36.5)	56.1 (43.6)	0.009^d^	<0.001	<0.001
RBDSQ	0.9 (0.9)	4.6 (2.8)	4.0 (2.8)	4.1 (3.1)	<0.001^d^	<0.001	<0.001
BDI-II	2.0 (2.0)	11.0 (6.9)	9.8 (6.4)	10.4 (7.1)	<0.001^d^	<0.001	<0.001
ESS	4.8 (2.8)	9.3 (4.9)	7.9 (4.8)	8.8 (6.2)	<0.001^d^	<0.001	<0.001
PDQ-39 summary index	1.2 (1.5)	11.4 (8.9)	19.4 (15.5)	24.5 (17.4)	<0.001^d^	<0.001	<0.001
QUIP	0.1 (0.4)	0.8 (1.4)	0.4 (0.9)	0.7 (1.7)	0.004^d^	0.164	0.066

Data represent the mean (standard deviation) or value (%).

*PD* Parkinson’s disease, *DLB* Dementia with Lewy bodies, *DaT* dopamine transporter, *MIBG* metaiodobenzylguanidine, *MoCA-J* the Japanese version of the Montreal Cognitive Assessment, *LEDD* Levodopa equivalent daily dose, *MDS-UPDRS* Movement Disorder Society-Unified Parkinson’s Disease Rating Scale, *SCOPA-AUT* the Japanese version of the Scale for Outcomes in Parkinson’s disease for Autonomic Symptoms, *SAOQ* Self-administered Odor Question, *RBDSQ* RBD screening scale, *BDI- II* Beck Depression Inventory-Second Edition, *ESS* Epworth Sleepiness Scale, *PDQ-39* Parkinson’s Disease Questionnaire-39, *QUIP* Questionnaire for Impulsive-Compulsive Disorders in Parkinson’s disease.

^a^*p* values determined by pairwise comparisons using Fisher’s exact test with Benjamini–Hochberg correction.

^b^*p* values determined by a one-way ANOVA with Tukey post-hoc test.

^c^*p* values determined by Fisher’s exact test.

^d^*p* values determined by analysis of covariance (ANCOVA) adjusted for age and sex with Tukey’s post-hoc test using the Benjamini–Hochberg method.

^e^Two patients with PD and three patients with DLB could not complete the Stroop test.

^f^One patient with DLB could not complete the line orientation test.

The PD-CI group participants were significantly older than the PD-CN group participants. The PD-CI group participants, who were selected based on the MoCA-J scores, had worse line orientation test scores than those of the PD-CN group participants. Compared with the PD-CN group participants, the PD-CI participants had worse RBDSQ, BDI-II, PDQ-39, and QUIP scores, while no significant difference was found in motor function (Supplementary Table 1).

### Plasma biomarkers

Pearson’s correlation analysis between plasma biomarkers and age exhibited a weak correlation for plasma Aβ composite in the PD group, weak correlations for plasma p-tau181 in the low-risk, high-risk, and PD groups, and moderate correlations for plasma NfL in the high-risk and PD groups (Supplementary Fig. 1). Therefore, all statistical tests regarding plasma biomarkers were adjusted for age. Considering the phenotypic differences of AD between females and males, biomarker values for sex were also adjusted^13^.

**Fig. 1:**
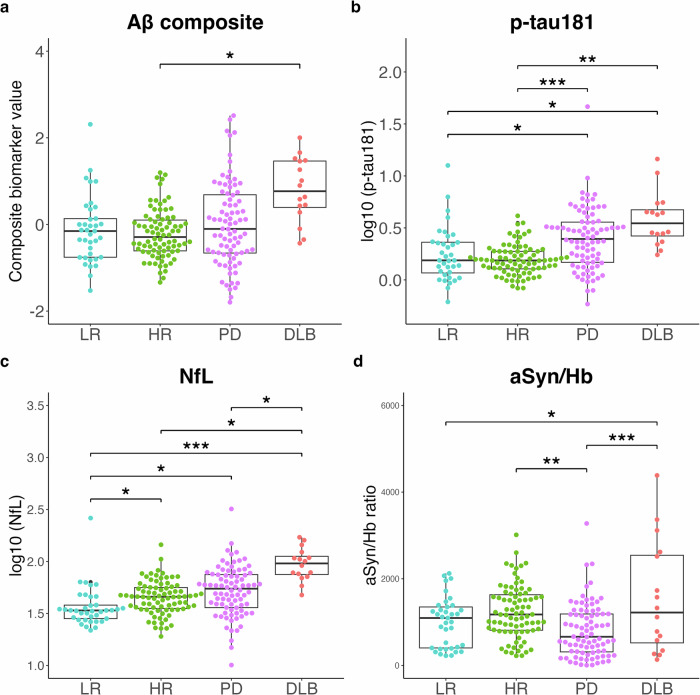
Levels of plasma biomarkers across diagnostic groups. Levels of Aβ composite (**a**), p-tau181 (**b**), NfL (**c**), and aSyn/Hb (**d**) are plotted with individual values and boxplots across diagnostic groups. Analysis of covariance (ANCOVA) adjusted for age and sex is used to determine *p* values visualized with ***p < 0.001, **p < 0.01, *p < 0.05. Aβ composite, a combination biomarker of amyloid-beta precursor protein (APP)_669-711_/amyloid-beta (Aβ)_1-42_ and Aβ_1-40_/Aβ_1-42_ ratios, p-tau181 phosphorylated tau 181, NfL neurofilament light chain, aSyn/Hb alpha-synuclein/hemoglobin ratio, LR low-risk, HR high-risk, PD Parkinson’s disease, DLB dementia with Lewy bodies.

Plasma Aβ composite levels in the DLB group were higher than those in the other groups; however, this difference was significant only between the DLB and the high-risk groups (Fig. 1a). Plasma log_10_ (p-tau181) levels were significantly higher in the PD and DLB groups than those in the low- and high-risk groups (Fig. 1b). Plasma log_10_ (NfL) levels were significantly higher in the high-risk, PD, and DLB groups than those in the low-risk group, with the DLB group exhibiting a pronounced elevation (Fig. 1c). Plasma aSyn/Hb ratios were significantly lower in the PD group than those in the high-risk and DLB groups. Plasma aSyn/Hb ratios were significantly higher in the DLB group than those in the low-risk group (Fig. 1d). Plasma aSyn levels, similar to aSyn/Hb ratios, were significantly lower in the PD group than those in the high-risk and DLB groups, and significantly higher in the DLB group than those in the low-risk group (Supplementary Fig. 2a). Hemoglobin levels were significantly lower in the PD group than those in the high-risk group and significantly lower in the DLB group than those in the low- and high-risk groups (Supplementary Fig. 2b).

**Fig. 2:**
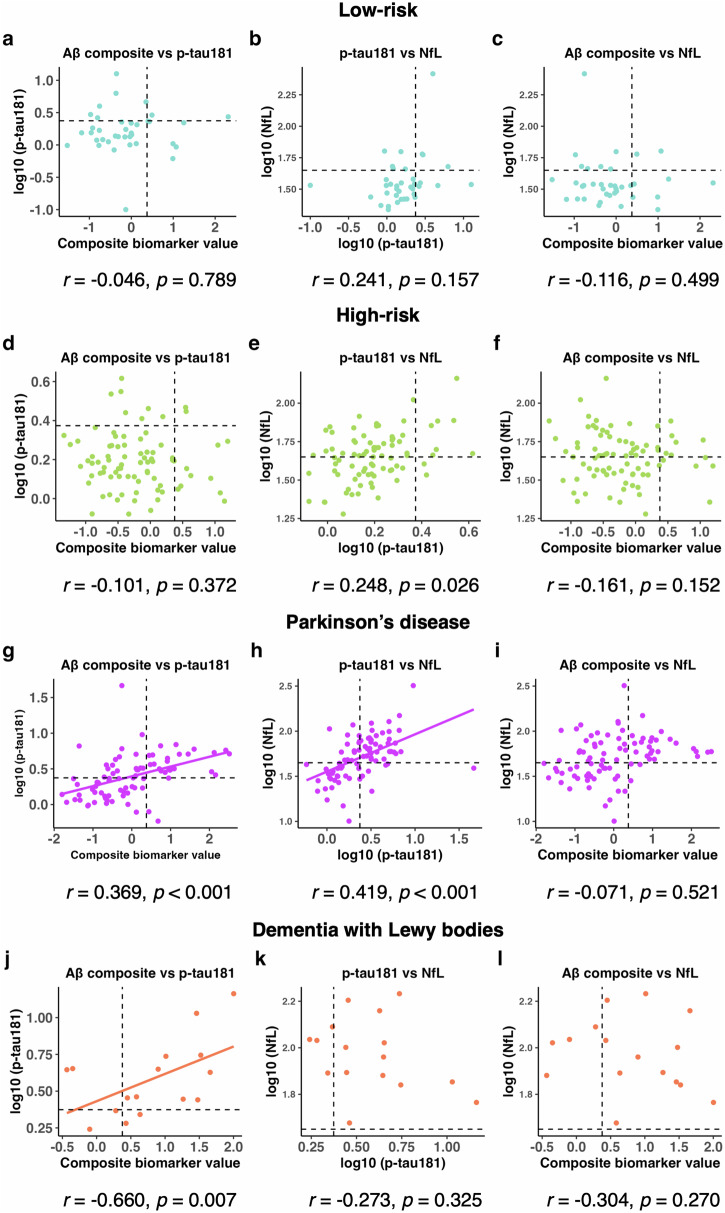
Age-adjusted partial correlation between plasma biomarkers. Age-adjusted Pearson’s partial correlation test among the plasma biomarkers in the low-risk group (**a**–**c**), high-risk group (**d**–**f**), Parkinson’s disease group (**g**–**i**), and dementia with Lewy bodies group (**j**–**l**). Cut-off values for Aβ composite, log_10_ (p-tau181), and log_10_ (NfL) are indicated by dotted lines (Aβ composite, 0.376; log_10_ [p-tau181], 0.374; log_10_ [NfL], 1.65). Aβ composite, combination biomarker of amyloid-beta precursor protein (APP)_669-711_/amyloid-beta (Aβ)_1-42_ and Aβ_1-40_/Aβ_1-42_ ratios; p-tau181, phosphorylated tau 181; NfL neurofilament light chain, aSyn/Hb alpha-synuclein/hemoglobin ratio.

The age-adjusted partial correlation analysis to assess the relationships between plasma biomarkers revealed no correlation between the plasma biomarkers in the low- and high-risk groups (Fig. 2a–f). In the PD group, Aβ composite and log_10_ (p-tau181) and log_10_ (p-tau181) and log_10_ (NfL) were weakly correlated (Fig. 2g–i). In the DLB group, Aβ composite and log_10_ (p-tau181) were moderately correlated (Fig. 2j–l).

**Fig. 3:**
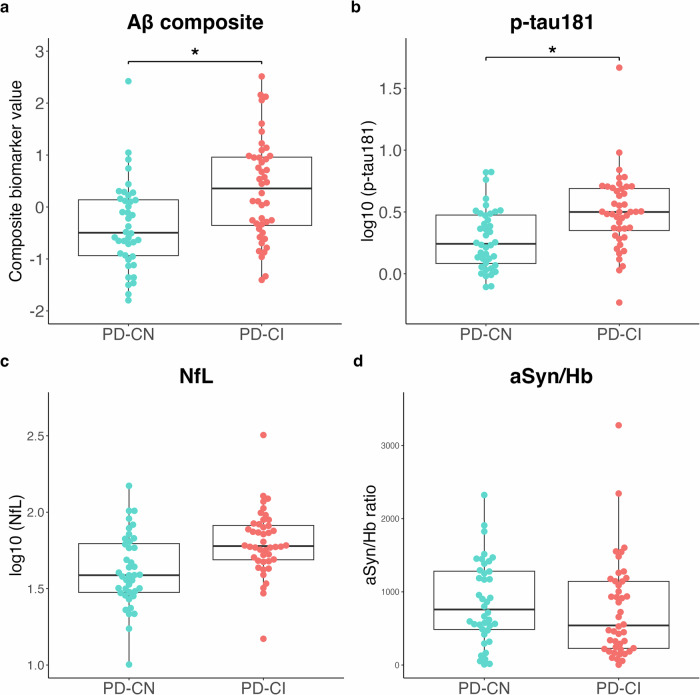
Subgroup analysis of plasma biomarkers by cognitive function in the Parkinson’s disease group. Levels of four plasma biomarkers (Aβ composite (**a**), p-tau181 (**b**), NfL (**c**), and aSyn/Hb (**d**)) are plotted with individual values and boxplots across diagnostic groups. The patients with PD with MoCA-J ≥ 26 and <26 are classified as cognitively normal (PD-CN) and cognitively impaired (PD-CI), respectively^27^. Analysis of covariance (ANCOVA) adjusted for age and sex is used to determine *p* values visualized with ***p < 0.001, **p < 0.01, *p < 0.05. Aβ composite combination biomarker of amyloid-beta precursor protein (APP)_669-711_/amyloid-beta (Aβ)_1-42_ and Aβ_1-40_/Aβ_1-42_ ratios, p-tau181 phosphorylated tau 181, NfL neurofilament light chain, aSyn/Hb alpha-synuclein/hemoglobin ratio, PD Parkinson’s disease, MoCA-J the Japanese version of the Montreal Cognitive Assessment.

The PD-CI group exhibited a significant increase in plasma Aβ composite and log_10_ (p-tau181) levels compared with the PD-CN group (Fig. 3a, b). Although plasma log_10_ (NfL) levels tended to be higher in the PD-CI group than in the PD-CN group, the difference was not significant (Fig. 3c). No significant differences were found in the aSyn/Hb ratio between the PD-CI and PD-CN groups (Fig. 3d).

### Differences in clinical features and plasma biomarkers of patients with PD and high-risk individuals across AT(N) profiles

Based on the cut-off values for Aβ composite, p-tau181, and NfL, participants were classified as normal/abnormal (−/+) for Aβ (A), tau (T), and neurodegeneration (N). The proportions of A − T − (N)− and A + T + (N)+ were significantly lower and higher, respectively, in the PD and DLB groups than those in the low-risk group. The proportion of A − T − (N)+ in the high-risk group was significantly higher than that in the low-risk group (Table 2).

**Table 2:** AT(N) profiles of the participants

AT(N) profiles	Low-risk (LR)	High-risk (HR)	PD	DLB	*p* values		
					LR vs HR	LR vs PD	LR vs DLB
A− T− (N)−, *n* (%)	19 (51.4)	30 (36.6)	25 (29.8)	0 (0.0)	0.192^a^	0.039	<0.001
A+ T− (N)−, *n* (%)	5 (13.5)	4 (4.9)	2 (2.4)	0 (0.0)	0.403^a^	0.164	0.614
A+ T+ (N)−, *n* (%)	1 (2.7)	0 (0.0)	1 (1.2)	0 (0.0)	1.000^a^	1.000	1.000
A+ T+ (N)+, *n* (%)	1 (2.7)	2 (2.4)	23 (27.4)	10 (62.5)	1.000^a^	0.002	<0.001
A+ T− (N)+, *n* (%)	1. (2.7)	5 (6.1)	1 (1.2)	2 (12.5)	0.664^a^	0.624	0.427
A− T+ (N)−, *n* (%)	3 (8.1)	2 (2.4)	5 (6.0)	0 (0.0)	1.000^a^	1.000	1.000
A− T− (N)+, *n* (%)	4 (10.8)	34 (41.5)	13 (15.5)	2 (12.5)	0.002^a^	0.873	1.000
A− T+ (N)+, *n* (%)	3 (8.1)	5 (6.1)	14 (16.7)	2 (12.5)	0.843^a^	0.640	0.843

Data represent value (%).

*PD* Parkinson’s disease, *DLB* Dementia with Lewy bodies.

A− Aβ composite < 0.376, A+ Aβ composite ≥ 0.376, T− log_10_ (p-tau181) < 0.374, T+ log_10_ (p-tau181) ≥ 0.374, N− log_10_ (NfL) < 1.65, N+ log_10_ (NfL) ≥ 1.65.

^a^*p* values determined by pairwise comparisons using Fisher’s exact test with Benjamini–Hochberg correction.

Patients with PD classified as A+ were older and had a shorter educational history, worse MoCA-J scores, and higher plasma log_10_ (p-tau181) levels than those classified as A−. Patients with PD classified as T+ were older and had worse Hoehn and Yahr Scale and MDS-UPDRS III total scores and subscores for bradykinesia and axial signs, and worse SCOPA-AUT, BDI-II, PDQ-39, and QUIP scores than those classified as T−. Patients with PD classified as T+ had higher levels of Aβ composite and log_10_ (NfL) than those classified as T−. Patients with PD classified as N+ were older and had worse scores on the MoCA-J and Hoehn and Yahr Scales, worse MDS-UPDRS III total scores and subscores for bradykinesia and axial signs, worse SCOPA-AUT, BDI-II, PDQ-39, and QUIP scores, and higher levels of plasma Aβ composite and log_10_ (p-tau181) than those classified as N− (Table 3).

**Table 3:** Clinical characteristics of the patients with Parkinson’s disease grouped by A/T/N profiles

	PD		*p* value	PD		*p* value	PD		*p* value
	A−	A+		T−	T+		N−	N+	
Number (M:F)	57 (31:26)	27 (13:14)	0.645^a^	41 (22:19)	43 (22:21)	0.831^a^	33 (17:16)	51 (27:24)	1.000^a^
Age, years	66.0 (9.2)	74.7 (6.6)	<0.001^b^	66.0 (9.0)	71.5 (9.1)	0.006^b^	63.2 (9.1)	72.5 (7.6)	<0.001^b^
Education, years	13.8 (2.6)	12.2 (3.6)	0.021^b^	13.5 (2.8)	13.0 (3.3)	0.444^b^	13.7 (2.4)	13.0 (3.4)	0.315^b^
DaT abnormal, %	33/33 (100)	10/10 (100)		24/24 (100)	19/19 (100)		18/18 (100)	25/25 (100)	
MIBG abnormal, %	28/34 (82.4)	13/14 (92.9)		20/23 (87.0)	21/25 (84.0)		17/21 (81.0)	24/27 (88.9)	
DaT and/or MIBG abnormal, %	43/43 (100)	19/19 (100)		32/32 (100)	30/30 (100)		27/27 (100)	35/35 (100)	
Disease duration, years	5.5 (4.1)	6.7 (6.4)	0.166^c^	5.3 (4.3)	6.5 (5.5)	0.170^c^	5.1 (3.6)	6.4 (5.6)	0.094^c^
MoCA-J	25.6 (3.1)	22.2 (4.7)	0.032^c^	25.7 (3.3)	23.4 (4.3)	0.109^c^	26.8 (2.4)	23.0 (4.1)	0.006^c^
Stroop test part 2 - part 1, sec	17.0 (13.4)^d^	32.4 (55.7)^d^	0.140^c^	14.4 (10.5)^d^	28.4 (45.2)^d^	0.129^c^	11.7 (8.4)	28.3 (41.9)^e^	0.079^c^
Line orientation test	16.0 (3.2)	15.3 (2.2)	0.863^c^	16.0 (3.4)	15.6 (2.5)	0.903^c^	16.6 (2.6)	15.3 (3.1)	0.349^c^
Hoehn and Yahr	2.0 (0.9)	2.5 (0.9)	0.075^c^	1.9 (0.8)	2.4 (1.0)	0.018^c^	1.8 (0.8)	2.4 (0.9)	0.013^c^
LEDD	409.6 (427.1)	389.1 (297.0)	0.496^c^	337.9 (306.2)	465.2 (447.9)	0.028^c^	312.5 (281.1)	461.6 (436.9)	0.002^c^
MDS-UPDRS III	23.7 (10.7)	27.7 (8.8)	0.259^c^	21.7 (8.9)	28.2 (10.6)	0.009^c^	20.0 (8.6)	28.3 (10.0)	<0.001^c^
Rigidity	3.6 (2.7)	3.5 (3.8)	0.332^c^	3.3 (2.6)	3.8 (3.5)	0.715^c^	3.1 (2.5)	3.9 (3.4)	0.681^c^
Tremor	3.2 (3.4)	5.0 (5.3)	0.151^c^	3.5 (4.0)	4.0 (4.4)	0.813^c^	3.3 (4.0)	4.0 (4.3)	0.988^c^
Bradykinesia	12.4 (5.9)	12.8 (4.8)	0.501^c^	11.2 (5.2)	13.8 (5.7)	0.014^c^	10.5 (4.7)	13.8 (5.7)	<0.001^c^
Axial signs	4.5 (3.9)	6.5 (3.3)	0.196^c^	3.7 (3.0)	6.5 (4.0)	0.004^c^	3.0 (2.7)	6.6 (3.7)	<0.001^c^
SCOPA-AUT	11.8 (7.7)	11.0 (9.7)	0.961^c^	9.3 (6.6)	13.7 (9.3)	0.004^c^	9.2 (5.4)	13.1 (9.6)	0.003^c^
SAOQ, %	68.0 (37.1)	67.7 (35.9)	0.902^c^	68.0 (38.5)	67.8 (35.0)	0.911^c^	66.1 (37.6)	69.1 (36.1)	0.426^c^
RBDSQ	4.0 (2.7)	3.9 (3.0)	0.634^c^	3.9 (2.7)	4.1 (2.9)	0.908^c^	3.6 (2.1)	4.2 (3.1)	0.598^c^
BDI-II	9.7 (5.8)	10.0 (7.5)	0.555^c^	7.9 (5.1)	11.5 (7.0)	0.002^c^	8.4 (5.5)	10.7 (6.8)	0.008^c^
ESS	8.4 (4.5)	6.9 (5.5)	0.915^c^	7.9 (4.5)	7.86 (5.24)	0.441^c^	8.6 (4.3)	7.5 (5.2)	0.811^c^
PDQ-39 summary index	19.7 (15.6)	18.7 (15.6)	0.900^c^	15.3 (14.2)	23.3 (15.9)	0.005^c^	14.5 (10.2)	22.5 (17.6)	0.001^c^
QUIP	0.4 (0.8)	0.5 (1.1)	0.069^c^	0.2 (0.6)	0.6 (1.1)	0.005^c^	0.2 (0.6)	0.5 (1.0)	0.004^c^
Aβ composite	−0.53 (0.56)	1.14 (0.61)	<0.001^c^	−0.57 (0.65)	0.56 (0.90)	<0.001^c^	−0.55 (0.65)	0.37 (0.98)	0.002^c^
log_10_ (p-Tau181)	0.33 (0.29)	0.54 (0.24)	0.026^c^	0.16 (0.15)	0.62 (0.22)	<0.001^c^	0.22 (0.33)	0.51 (0.20)	<0.001^c^
log_10_ (NfL)	1.67 (0.26)	1.81 (0.14)	0.748^c^	1.60 (0.23)	1.83 (0.19)	<0.001^c^	1.48 (0.14)	1.87 (0.15)	<0.001^c^
aSyn, pg/ml	11665.2 (8920.0)	9942.2 (8392.1)	0.625^c^	12083.8 (10187.0)	10184.2 (7134.9)	0.858^c^	11603.1 (8905.1)	10793.2 (8706.8)	0.301^c^
aSyn/Hb ratio	841.7 (640.60)	725.2 (570.7)	0.824^c^	844.5 (704.7)	765.9 (527.9)	0.935^c^	793.4 (577.3)	811.3 (648.5)	0.182^c^

Data represent the mean (standard deviation) or value (%).

*PD* Parkinson’s disease, *DaT* dopamine transporter, *MIBG* metaiodobenzylguanidine, *MoCA-J* the Japanese version of the Montreal Cognitive Assessment, *LEDD* Levodopa equivalent daily dose, *MDS-UPDRS* Movement Disorder Society-Unified Parkinson’s Disease Rating Scale, *SCOPA-AUT* the Japanese version of the Scale for Outcomes in Parkinson’s disease for Autonomic Symptoms, *SAOQ* Self-administered Odor Question, *RBDSQ* RBD screening scale, *BDI-II* Beck Depression Inventory-Second Edition, *ESS* Epworth Sleepiness Scale, *PDQ-39* Parkinson’s Disease Questionnaire-39, *QUIP* Questionnaire for Impulsive-Compulsive Disorders in Parkinson’s disease, *Aβ* amyloid-beta, *p-tau181* phosphorylated tau 181, *NfL* neurofilament light chain, *aSyn* alpha-synuclein.

A− Aβ composite < 0.376, A+ Aβ composite ≥ 0.376, T− log_10_ (p-tau181) < 0.374, T+ log_10_ (p-tau181) ≥ 0.374, N− log_10_ (NfL) < 1.65, N+, log_10_ (NfL) ≥ 1.65.

^a^*p* values determined by Fisher’s exact test.

^b^*p* values determined by Student’s *t* test.

^c^*p* values determined by analysis of covariance (ANCOVA) adjusted for age and sex.

^d^One patient with PD could not complete the Stroop test.

^e^Two patients with PD could not complete the Stroop test.

The age-adjusted partial correlation analysis that assessed the relationships between plasma biomarkers and clinical indices (Supplementary Fig. 3) revealed that plasma log_10_ (NfL) was weakly correlated with MDS-UPDRS III bradykinesia and axial signs subscores and the SCOPA-AUT and PDQ-39 scores. In addition, plasma log_10_ (p-tau181) was weakly correlated with the SCOPA-AUT and PDQ-39 scores, while plasma aSyn/Hb ratio was weakly correlated with the MDS-UPDRS III rigidity subscore.

No significant differences in age, cognitive and motor functions, questionnaire survey scores, or plasma biomarkers were found between the high-risk participants classified as A+ and A−. The high-risk participants classified as T+ were significantly older and had worse scores on the Hoehn and Yahr Scale, MDS-UPDRS III rigidity subscore, and the RBDSQ, and QUIP scales than those classified as T−. The high-risk participants classified as N+ were significantly older and had a higher rate of MIBG abnormalities than those classified as N− (Table 4). The age-adjusted partial correlation analysis revealed no significant correlations between plasma biomarkers and each clinical score (Supplementary Fig. 4).

**Table 4:** Clinical characteristics of the high-risk subjects grouped by A/T/N profiles

	High-risk		*p* value	High-risk		*p* value	High-risk		*p* value
	A−	A+		T−	T+		N−	N+	
Number (M:F)	71 (44:27)	11 (9:2)	0.313^a^	73 (47:26)	9 (6:3)	1.000^a^	36 (22:14)	46 (31:15)	0.644^a^
Age, years	64.3 (7.3)	68.5 (8.9)	0.090^b^	64.1 (7.3)	71.3 (7.1)	0.006^b^	61.3 (7.2)	67.7 (6.7)	<0.001^b^
Education, years	13.4 (2.1)	14.5 (2.5)	0.116^b^	13.6 (2.0)	13.1 (2.9)	0.553^b^	13.2 (1.7)	13.7 (2.4)	0.279^b^
DaT SBR average	6.50 (1.39)	6.37 (2.12)	0.854^c^	6.48 (1.22)	6.56 (3.06)	0.547^c^	6.58 (1.25)	6.41 (1.67)	0.876^c^
DaT Asymmetry Index	5.12 (3.60)	6.31 (7.32)	0.496^c^	5.45 (4.36)	3.88 (2.87)	0.185^c^	5.18 (3.46)	5.37 (4.80)	0.889^c^
DaT abnormal, %	16/71 (22.5)	5/11 (45.5)	0.139^a^	17/73 (23.3)	4/9 (44.4)	0.224^a^	9/36 (25.0)	12/46 (26.1)	1.000^a^
MIBG early	2.95 (0.63)	2.79 (0.54)	0.685^c^	2.92 (0.59)	3.04 (0.84)	0.318^c^	2.99 (0.58)	2.89 (0.65)	0.867^c^
MIBG delay	3.16 (0.91)	2.94 (0.78)	0.880^c^	3.14 (0.87)	3.02 (1.08)	0.721^c^	3.32 (0.78)	2.97 (0.95)	0.402^c^
MIBG washout ratio, %	22.59 (19.52)	21.47 (19.55)	0.291^c^	21.34 (18.93)	31.31 (22.13)	0.658^c^	16.74 (14.17)	26.90 (21.81)	0.322^c^
MIBG abnormal, %	13/71 (18.3)	2/11 (18.2)	1.000^a^	13/73 (17.8)	2/9 (22.2)	0.666^a^	2/36 (5.6)	13/46 (28.3)	0.018^a^
DaT and/or MIBG abnormal, %	25/71 (35.2)	5/11 (45.5)	0.520^a^	26/73 (35.6)	4 /9 (44.4)	0.718^a^	10/36 (27.8)	20/46 (43.5)	0.170^a^
MoCA-J	26.6 (3.1)	27.4 (1.4)	0.076^c^	26.8 (2.9)	25.6 (2.9)	0.929^c^	26.9 (2.2)	26.4 (3.4)	0.350^c^
Stroop test part 2 - part 1, sec	12.2 (8.6)	17.3 (8.0)	0.554^c^	12.4 (8.7)	15.4 (7.6)	0.928^c^	10.5 (6.7)	14.5 (9.5)	0.546^c^
Line orientation test	17.0 (3.1)	17.2 (1.7)	0.494^c^	17.1 (2.8)	16.2 (3.4)	0.929^c^	17.5 (2.7)	16.6 (3.0)	0.917^c^
MDS-UPDRS III	4.4 (4.4)	4.6 (2.1)	0.373^c^	4.0 (3.8)	7.6 (5.4)	0.200^c^	3.3 (3.0)	5.3 (4.7)	0.744^c^
Rigidity	0.4 (0.8)	0.4 (0.5)	0.298^c^	0.3 (0.7)	1.1 (0.9)	0.012^c^	0.3 (0.5)	0.5 (0.8)	0.726^c^
Tremor	0.4 (1.0)	0.7 (0.8)	0.586^c^	0.5 (1.0)	0.4 (0.7)	0.466^c^	0.3 (0.6)	0.6 (1.1)	0.743^c^
Bradykinesia	2.4 (2.5)	1.9 (1.5)	0.163^c^	2.2 (2.3)	3.6 (3.2)	0.549^c^	1.9 (2.1)	2.7 (2.6)	0.966^c^
Axial signs	1.2 (1.5)	1.6 (1.0)	0.940^c^	1.1 (1.2)	2.4 (2.7)	0.077^c^	0.9 (1.0)	1.5 (1.6)	0.668^c^
OSIT-J	8.8 (2.9)	8.7 (1.4)	0.489^c^	9.0 (2.6)	7.2 (3.4)	0.261^c^	8.9 (3.1)	8.7 (2.4)	0.333^c^
CVRR rest, %	3.20 (1.71)	3.14 (1.46)	0.871^c^	3.1 (1.5)	3.7 (2.7)	0.135^c^	3.2 (1.5)	3.2 (1.8)	0.596^c^
SCOPA-AUT	9.8 (4.8)	13.0 (5.7)	0.060^c^	10.0 (5.1)	12.2 (4.2)	0.174^c^	9.3 (4.7)	10.9 (5.3)	0.117^c^
SAOQ, %	81.8 (27.1)	92.8 (12.9)	0.086^c^	84.8 (24.5)	70.9 (34.4)	0.214^c^	84.0 (25.7)	82.7 (26.2)	0.756^c^
RBDSQ	4.6 (2.9)	4.6 (2.5)	0.980^c^	4.4 (2.8)	6.4 (2.4)	0.022^c^	5.2 (2.9)	4.1 (2.7)	0.055^c^
BDI-II	10.9 (7.2)	11.2 (5.1)	0.646^c^	11.0 (7.1)	11.00 (5.7)	0.771^c^	12.3 (7.1)	9.9 (6.6)	0.224^c^
ESS	9.3 (5.1)	8.8 (4.1)	0.986^c^	9.2 (4.8)	9.8 (6.1)	0.233^c^	10.7 (4.9)	8.1 (4.6)	0.124^c^
PDQ-39 summary index	11.7 (9.3)	9.1 (4.7)	0.591^c^	11.3 (9.1)	12.4 (6.8)	0.348^c^	13.5 (11.4)	9.7 (5.8)	0.206^c^
QUIP	0.8 (1.4)	1.0 (1.6)	0.679^c^	0.7 (1.1)	1.9 (2.7)	0.006^c^	1.1 (1.7)	0.6 (1.1)	0.155^c^
Aβ composite	-0.38 (0.41)	0.78 (0.30)	<0.001^c^	−0.24 (0.57)	−0.08 (0.44)	0.507^c^	−0.19 (0.56)	−0.25 (0.57)	0.467^c^
log_10_ (p-Tau181)	0.20 (0.14)	0.18 (0.17)	0.239^c^	0.16 (0.11)	0.48 (0.07)	<0.001^c^	0.15 (0.12)	0.22 (0.16)	0.489^c^
log_10_ (NfL)	1.65 (0.16)	1.67 (0.14)	0.440^c^	1.64 (0.15)	1.77 (0.19)	0.238^c^	1.51 (0.09)	1.76 (0.11)	<0.001^c^
aSyn, pg/ml	17613.0 (9048.4)	20828.5 (9169.9)	0.355^c^	18205.8 (9524.1)	16734.9 (3907.3)	0.684^c^	18774.6 (9038.5)	17472.9 (9161.5)	0.489^c^
aSyn/Hb ratio	1213.1 (613.1)	1412.8 (561.8)	0.373^c^	1244.5 (634.1)	1202.3 (333.5)	0.832^c^	1279.8 (596.9)	1208.7 (619.6)	0.526^c^

Data represent the mean (standard deviation) or value (%).

*DaT* dopamine transporter, *MIBG* metaiodobenzylguanidine, *MoCA-J* the Japanese version of the Montreal Cognitive Assessment, *MDS-UPDRS* Movement Disorder Society-Unified Parkinson’s Disease Rating Scale, *OSIT-J* the odor stick identification test for Japanese, *CVRR* coefficient of variation of RR intervals, *SCOPA-AUT* the Japanese version of the Scale for Outcomes in Parkinson’s disease for Autonomic Symptoms, *SAOQ* Self-administered Odor Question, *RBDSQ* RBD screening scale, *BDI-II* Beck Depression Inventory-Second Edition, *ESS* Epworth Sleepiness Scale, *PDQ-39* Parkinson’s Disease Questionnaire-39, *QUIP* Questionnaire for Impulsive-Compulsive Disorders in Parkinson’s disease, *Aβ* amyloid-beta, *p-tau181* phosphorylated tau 181, *NfL* neurofilament light chain, *aSyn* alpha-synuclein.

A− Aβ composite < 0.376, A+ Aβ composite ≥ 0.376, T− log_10_ (p-tau181) <0.374, T+ log_10_ (p-tau181) ≥0.374, N− log_10_ (NfL) <1.65, N+ log_10_ (NfL) ≥1.65.

^a^*p* values determined by Fisher’s exact test.

^b^*p* values determined by Student’s *t* test.

^c^*p* values determined by analysis of covariance (ANCOVA) adjusted for age and sex.

## Discussion

This study measured and analyzed plasma Aβ composite, p-tau181, NfL, and aSyn in patients with PD and DLB and high- and low-risk individuals who were identified in a questionnaire survey on prodromal symptoms of LBD. The results revealed that both PD and DLB groups had increased plasma p-tau181 levels, indicating that comorbid AD neuropathology exists in manifest LBD. In addition, plasma NfL levels were elevated in the high-risk group despite the absence of significant elevation in AD-related plasma biomarker levels such as Aβ composite and p-tau181; thus, plasma NfL levels may reflect aSyn-induced neurodegeneration in the prodromal phase of LBD.

Previous studies demonstrated that higher plasma Aβ composite levels can predict Aβ burden with approximately 90% accuracy when using Pittsburgh compound-B (PIB)-amyloid positron emission tomography (PET) as the standard of truth^6^, and higher plasma p-tau181 levels can predict Aβ and tau positivity on PET^7^. In the present study, although plasma Aβ composite levels were higher in the DLB group than those in the other groups, the difference was only significant between the DLB and the high-risk groups, unlike the increase in plasma p-tau181 levels which was significant in both the PD and DLB groups. Although this incongruity may be a result of the limited statistical power in the multigroup comparison, previous PET studies reported a substantially low incidence of amyloid deposition in PD without dementia^14,15^. Another plasma biomarker study reported that the plasma Aβ_1-42_/Aβ_1-40_ ratio was increased in the PD without dementia group compared with that in healthy controls and decreased in the PD with dementia group compared with that in the PD without dementia group^9^. Collectively, these findings suggest that amyloid pathology develops concurrently with cognitive decline in LBD and that p-tau biomarkers are more sensitive than Aβ biomarkers in early PD.

However, in our focused analysis on the PD group, both AD-related plasma biomarker (Aβ composite and p-tau181) levels were significantly higher in the PD-CI group than those in the PD-CN group. This suggests that comorbid AD neuropathology influences the development of cognitive impairment in PD, consistent with previous cerebrospinal fluid (CSF) studies^16^. In the patients with PD, those classified as A+ had worse MoCA-J scores compared with those classified as A−, and those classified as T+ had worse scores on motor function (MDS-UPDRS III subscores on bradykinesia and axial signs), the SCOPA-AUT, PDQ-39, and QUIP scales compared with those classified as T−. These observations are consistent with a previous CSF study which reported that lower CSF Aβ_1-42_ and higher p-tau were associated with delayed memory recall and motor function, respectively^17^.

Conversely, the AD-related plasma biomarkers were not elevated in the high-risk group. These results are consistent with those reported in previous studies, namely, that the rate of positive amyloid PET in patients with idiopathic RBD was similar to that in cognitively normal individuals^18–20^. Although these findings indicate that AD-related plasma biomarkers become detectable after the manifestation of motor/cognitive symptoms in LBD, comorbid AD neuropathology may subsist at an undetectable level in the prodromal phase and influence disease progression. Therefore, further longitudinal data analysis is required to elucidate the role of AD-related plasma biomarkers on motor function and non-motor symptoms in high-risk individuals.

Elevated plasma NfL level is a reliable biomarker of neurodegeneration in various diseases^8^. Although results of previous cross-sectional studies on PD on the correlation between plasma NfL levels and cognitive and motor functions are inconclusive, those of prospective studies consistently demonstrate a correlation between baseline plasma NfL and worsening cognitive and motor functions^21^. Another study reported that higher baseline NfL levels in patients with idiopathic RBD were associated with worsening cognitive, motor, and autonomic functions and a higher risk of phenoconversion^11^. The present study demonstrated that plasma NfL levels were significantly elevated in the PD, DLB, and high-risk groups compared with those in the low-risk group. In patients with PD, those classified as N+ had worse scores on the MoCA-J, Hoehn and Yahr, MDS-UPDRS III, SCOPA-AUT, BDI-II, PDQ-39, and QUIP scales than patients classified as N−, suggesting that plasma NfL levels are related to cognitive function, and motor and non-motor symptoms. In the high-risk participants, although no significant differences were observed in cognitive function, or motor or non-motor symptoms between those classified as N+ and N−, participants classified as N+ had a higher rate of abnormal MIBG findings than those classified as N−, suggesting that plasma NfL indicates aSyn-induced neurodegeneration, particularly its peripheral involvement, at the prodromal phase. However, as NfL levels may be elevated in various neurodegenerative diseases, longitudinal observations are necessary to confirm phenoconversion and ascertain that the NfL elevation in the high-risk group is indeed caused by aSyn pathology.

Previous studies demonstrated that CSF aSyn is decreased in patients with PD^22^. However, results for studies on plasma aSyn levels have been inconsistent, possibly because plasma aSyn levels can be affected by contamination with red blood cells in which aSyn is abundant^23^. In the present study, we attempted to correct aSyn for hemoglobin levels. The plasma aSyn/Hb ratio was significantly decreased in the PD group compared with that in the high-risk and DLB groups and significantly elevated in the DLB group compared with that in the low-risk group. This inconsistent result indicates that plasma aSyn measurement via Simoa may have limitations, and techniques, such as real-time quaking-induced conversion (RT-QUIC), may be necessary^24^.

This study has some limitations. First, the sample size was small, and discrepancies in age and sex ratio among the groups were present. Therefore, the results of this study may not be generalizable and need to be validated with a larger sample. Second, high-risk participants were selected based on a questionnaire survey on prodromal symptoms, and phenoconversion is yet to be confirmed. Further longitudinal studies are needed to confirm the precise risk of developing LBD in these individuals. Third, diagnoses of PD and DLB were based on clinical evaluations rather than neuropathological confirmation. Fourth, AT(N) profile was determined only by plasma biomarkers, and no PET or CSF studies were performed. Moreover, given the lack of neuropathological evaluations, attributing the changes in AD-related plasma biomarkers solely to AD pathology may overestimate the specificity of these biomarkers. Fifth, the cross-sectional nature of our study limits our ability to establish causal relationships or determine the temporal sequence of biomarker changes and symptom onset. Longitudinal studies are needed to achieve better understanding of the biomarker change in the prodromal phase of LBD.

In conclusion, our study demonstrated that comorbid AD neuropathology is present at the symptomatic phase of LBD. In PD, plasma Aβ composite was associated with general cognitive function, plasma p-tau181 with motor function and non-motor symptoms, and plasma NfL with cognitive and motor functions and non-motor symptoms. In addition, the elevated plasma NfL levels in the high-risk group, despite the absence of changes in AD-related plasma biomarkers, suggested the potential of plasma NfL as a biomarker to detect aSyn-induced neurodegeneration in the prodromal phase of LBD.

## Methods

### Study design and participants

The Nagoya-Takayama preclinical/prodromal Lewy body disease (NaT-PROBE) study is a prospective, longitudinal, multi-center, community-based cohort study coordinated by the Nagoya University School of Medicine. Between March 2017 and January 2023, healthy individuals aged ≥50 years who visited the Kumiai Kosei Hospital, Daido Clinic, or Chutoen General Medical Center, in Japan, for their annual health checkup were surveyed using the following questionnaires: the Japanese version of the Scale for Outcomes in Parkinson’s disease for Autonomic Symptoms (SCOPA-AUT); the Self-administered Odor Question (SAOQ); the RBD screening scale (RBDSQ); the Beck Depression Inventory-Second Edition (BDI-II); the Epworth Sleepiness Scale (ESS); and the Physical Activity Scale for the Elderly (PASE)^3^. Based on the results of our previous study^2^, 82 and 37 consecutive participants with ≥2 abnormal scores (high-risk group) and no abnormalities (low-risk group), respectively, in the SCOPA-AUT, SAOQ, and RBDSQ scales were enrolled in the present study. The cut-off value for identifying the high-risk group was 10, 90.0%, and 5 for the SCOPA-AUT, SAOQ, and RBDSQ scales, respectively^2^.

In addition, patients with PD and DLB who visited Nagoya University Hospital, Kumiai Kosei Hospital, and the National Center for Geriatrics and Gerontology between March 2017 and January 2023 were evaluated. Among these, 84 patients with PD, who met the United Kingdom Parkinson’s Disease Society Brain Bank Diagnostic Criteria^25^, and 14 patients with DLB, who met the diagnostic criteria of the fourth report of the DLB consortium^26^, were enrolled in the present study.

Comprehensive evaluations, including cognitive and motor function assessments, questionnaire surveys, and blood sampling were conducted for all participants. Additionally, DaT-SPECT and cardiac MIBG scintigraphy were performed for all high- and low-risk participants.

### Cognitive and motor function examination

The Japanese version of the Montreal Cognitive Assessment (MoCA-J), the Stroop test, and the line orientation test were used to assess the general cognitive, frontal lobe, and visuospatial cognitive functions, respectively. Patients with PD with MoCA-J ≥ 26 and <26 were classified as cognitively normal (PD-CN) and cognitively impaired (PD-CI), respectively, according to previously proposed criteria^27^. The Movement Disorder Society-Unified Parkinson’s Disease Rating Scale (MDS-UPDRS) was scored by neurologists who were certified MDS-UPDRS evaluators for assessing PD-related motor and non-motor symptoms. Rigidity (3.3), tremor (3.15–3.18), bradykinesia (3.2, 3.4–3.8, and 3.14), and axial signs (3.1 and 3.9–3.13) scores were extracted from the MDS-UPDRS III for further analysis. Levodopa equivalent daily dose (LEDD) was calculated as previously described^28^.

### Questionnaires on motor and non-motor symptoms

The SCOPA-AUT (Japanese version), SAOQ, RBDSQ, BDI-II, ESS, Parkinson’s Disease Questionnaire-39 (PDQ-39), and Questionnaire for Impulsive-Compulsive Disorders in Parkinson’s Disease (QUIP) were used to evaluate autonomic dysfunction, olfactory dysfunction, RBD, depressive symptoms excessive daytime sleepiness, PD-specific health-related quality of life, and impulse control disorder, respectively. All the aforementioned questionnaires were validated for self-administration in a Japanese population^29–35^.

### Imaging tests

DaT-SPECT imaging with (^123^I)FP-CIT and cardiac (^123^I)MIBG scintigraphy (^123^I-MIBG) were performed to detect presynaptic dopamine neuronal dysfunction and to assess postganglionic cardiac autonomic denervation, respectively. DaT-SPECT and MIBG scintigraphy were measured as previously described^3^. DaT-SPECT was considered abnormal when decreased DaT-SPECT Specific Binding Ratio (SBR) or abnormal visual findings were observed. The reference values of Japanese volunteers were used to evaluate the decrease in DaT SPECT SBR^36^. MIBG was considered abnormal when early or delayed H/M ratios were <2.2^37^.

### Sample collection and plasma biomarker measurements

Plasma samples, collected in EDTA-2Na-containing tubes, were centrifuged for 10 min at 1200 or 3000 × *g*, aliquoted, and immediately stored at −80 °C. Plasma Aβ composite was measured via immunoprecipitation-mass spectrometry (IP-MS) assays as previously described^6^. Plasma p-tau181, NfL, and aSyn were measured by a single-molecule array (Simoa) using pTau-181 Advantage V2 Kit, NF-light Advantage V2 Kit, and Alpha-Synuclein Discovery Kit (Quanterix, Billerica, MA, USA). Hemoglobin was measured by sodium lauryl sulfate (SLS) hemoglobin detection method.

The cut-off value for plasma Aβ composite was set at 0.376, based on previous studies^6,38^. This value was calculated using PIB-PET as the standard of truth from two cohorts: the Japanese National Center for Geriatrics and Gerontology (NCGG) and the Australian Imaging, Biomarker and Lifestyle Study of Aging (AIBL)^6^. The distribution of the Aβ composite levels was largely consistent between these historical cohorts used to establish the cut-off value and our current cohort (Supplementary Fig. 5). The plasma p-tau181 and NfL levels were log-transformed with base 10 to approximate a normal distribution, and the 95% Confidence Interval (CI) upper limit of the low-risk participants without abnormal plasma Aβ composite and DaT-SPECT and MIBG imaging was used for cut-off values (log_10_ [p-tau181], 0.374; log_10_ [NfL], 1.65). These cut-off values were used to determine the AT(N) profile^39^ (A−, Aβ composite < 0.376; A+, Aβ composite ≥ 0.376; T−, log_10_ [p-tau181] < 0.374; T+, log_10_ [p-tau181] ≥ 0.374; N−, log_10_ [NfL] <1.65; N+, log_10_ [NfL] ≥ 1.65).

### Statistical analyses

All data represented the mean (standard deviation), unless otherwise stated. Since aSyn is abundant in red blood cells^23^, the aSyn/Hb ratio, corrected using hemoglobin levels, was used in the analysis. The demographic scores of the low-risk, high-risk, PD, and DLB groups were compared using a parametric one-way analysis of variance (ANOVA), followed by Tukey’s test. Between-group categorical variables were compared using Fisher’s exact test. The Benjamini–Hochberg method was used for multiple comparisons. The clinical scores and plasma biomarkers of the low-risk, high-risk, PD, and DLB groups were compared using the analysis of covariance (ANCOVA) adjusted for age and sex, followed by Tukey’s test using the Benjamini–Hochberg method. When comparing A+ and A−, T+ and T−, and N+ and N− among the patients with PD or high-risk participants, Student’s *t* test was used for the demographic scores, and ANCOVA adjusted for age and sex was used for the clinical scores and plasma biomarkers. Pearson’s correlation coefficient was used to determine the relationship between plasma biomarkers and age. Age-adjusted Pearson’s partial correlation coefficient was used to determine the relationships between the plasma biomarkers and between the plasma biomarkers and each clinical score.

*p* values < 0.05 were considered statistically significant. Correlation coefficients (*r*) were interpreted as follows: >0.8, ‘very strong’; 0.5–0.8, ‘moderately strong’; and 0.3–0.5, ‘weak’. All statistical analyses were performed using R version 4.2.0, R Foundation for Statistical Computing, Vienna, Austria (https://www.R-project.org/). Figures were generated using the R package *ggplot2*.

### Supplementary information

Supplemental Material

## Data Availability

The data used to support the findings of this study are available from the corresponding author upon reasonable request, which includes the specification of a clear research question and confirmation of the approval from the Ethics Review Committee of Nagoya University Graduate School of Medicine.
