# Supplementary material for: Plasma biomarkers of neurodegeneration in patients and high risk subjects with Lewy body disease
Source: NPJ Parkinsons Dis. 2024 Jul 31;10:135. doi: 10.1038/s41531-024-00745-8 (PMC11292020; doi:10.1038/s41531-024-00745-8)
Supplement: Supplementary file 1 — Supplemental Material [file 41531_2024_745_MOESM1_ESM.pdf]

Supplemental material

Plasma biomarkers of neurodegeneration in patients and high risk subjects with Lewy body disease

Keita Hiraga, Makoto Hattori, Yuki Satake, Daigo Tamakoshi, Taiki Fukushima, Takashi Uematsu, Takashi Tsuboi, Maki Sato, Katsunori Yokoi, Keisuke Suzuki, Yutaka Arahata, Yukihiko Washimi, Akihiro Hori, Masayuki Yamamoto, Hideaki Shimizu, Masakazu Wakai, Harutsugu Tatebe, Takahiko Tokuda, Akinori Nakamura, Shumpei Niida, Masahisa Katsuno

Contents:

Supplementary Table: 1

Supplementary Figures: 5

Supplementary Table 1. Background characteristics of the patients with Parkinson's disease by cognitive function

	PD-CN	PD-CI	*p* value
Number (M:F)	40 (22:18)	44 (22:22)	0.668[a]
Age, years	64.4 (9.0)	72.8 (7.8)	< 0.001[b]
Education, years	13.6 (2.4)	12.9 (3.5)	0.284[b]
DaT abnormal, %	23/23 (100)	20/20 (100)	1.000[a]
MIBG abnormal, %	21/26 (80.8)	20/22 (90.9)	0.429[a]
DaT and/or MIBG abnormal, %	33/33 (100)	29/29 (100)	1.000[a]
Disease duration, years	6.3 (5.0)	5.5 (4.9)	0.582[c]
MoCA-J	27.6 (1.3)	21.7 (3.5)	< 0.001[c]
Stroop test part 2 - part 1, sec	12.8 (7.5)	30.0 (45.2)*	0.062[c]
Line orientation test	17.2 (2.1)	14.5 (3.0)	< 0.001[c]
Hoehn and Yahr	1.9 (0.8)	2.3 (1.0)	0.222[c]
LEDD	423.6 (449.6)	384.3 (327.1)	0.654[c]
MDS-UPDRS III	22.6 (9.8)	27.2 (10.3)	0.121[c]
Rigidity	3.2 (2.5)	3.9 (3.5)	0.701[c]
Tremor	3.6 (4.0)	3.9 (4.4)	0.754[c]
Bradykinesia	11.8 (5.4)	13.2 (5.7)	0.093[c]
Axial signs	4.0 (3.4)	6.2 (3.8)	0.074[c]
SCOPA-AUT	10.5 (7.0)	12.5 (9.4)	0.077[c]
SAOQ, %	65.1 (38.3)	70.4 (35.1)	0.343[c]
RBDSQ	3.3 (2.1)	4.6 (3.2)	0.034[c]
BDI-II	8.8 (5.8)	10.7 (6.8)	0.045[c]
ESS	8.0 (4.6)	7.8 (5.1)	0.205[c]
PDQ-39 summary index	15.8 (11.5)	22.7 (17.9)	0.005[c]
QUIP	0.3 (0.8)	0.5 (1.0)	0.038[c]

Patients with PD with MoCA-J ≥ 26 and < 26 are classified as cognitively normal (PD-CN) and cognitively impaired (PD-CI), respectively

PD, Parkinson's disease; DaT, dopamine transporter; MIBG, metaiodobenzylguanidine; MoCA-J, the Japanese version of the Montreal Cognitive Assessment; LEDD, Levodopa equivalent daily dose; MDS-UPDRS, Movement Disorder Society-Unified Parkinson's Disease Rating Scale; SCOPA-AUT, the Japanese version of the Scale for Outcomes in Parkinson's disease for Autonomic Symptoms; SAOQ, Self-administered Odor Question; RBDSQ, RBD screening scale; BDI-II, Beck Depression Inventory-Second

Edition; ESS, Epworth Sleepiness Scale; PDQ-39, Parkinson's Disease Questionnaire-39; QUIP, Questionnaire for Impulsive-Compulsive Disorders in Parkinson's disease

*Two patients with PD patients could not complete the Stroop test

[a]*p* values determined by Fisher's exact test

[b]*p* values determined by Student's *t*-test

[c]*p* values determined by analysis of covariance (ANCOVA) adjusted for age and sex

Data represent the mean (standard deviation) or value (%)

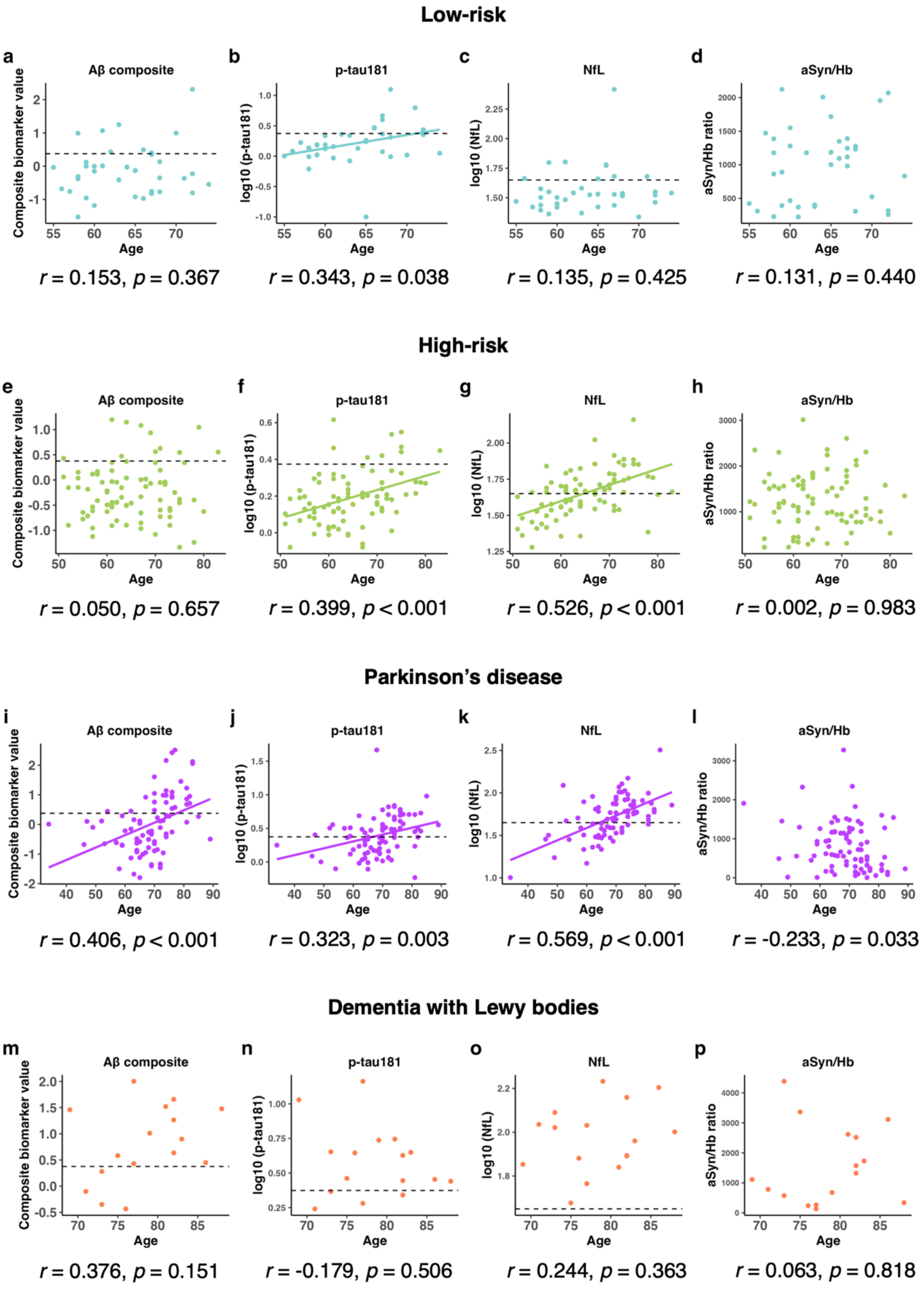

Supplementary Figure 1. Pearson's correlation test between plasma biomarkers and age in the low-risk group (a–d), high-risk group (e–h), Parkinson's disease group (i–l), and dementia with Lewy bodies group (m–p). The cut-off values for Aβ composite, $\log_{10}$ (p-tau181), and $\log_{10}$ (NfL) are indicated by dotted lines (Aβ composite, 0.376; $\log_{10}$ [p-tau181], 0.374; $\log_{10}$

[NfL], 1.65). Aβ composite, combination biomarker of amyloid-beta precursor protein $(APP)_{669\text{-}711}$/amyloid-beta $(Aβ)_{1\text{-}42}$ and $Aβ_{1\text{-}40}/Aβ_{1\text{-}42}$ ratios; p-tau181, phosphorylated tau 181; NfL, neurofilament light chain; aSyn/Hb, alpha-synuclein/hemoglobin ratio

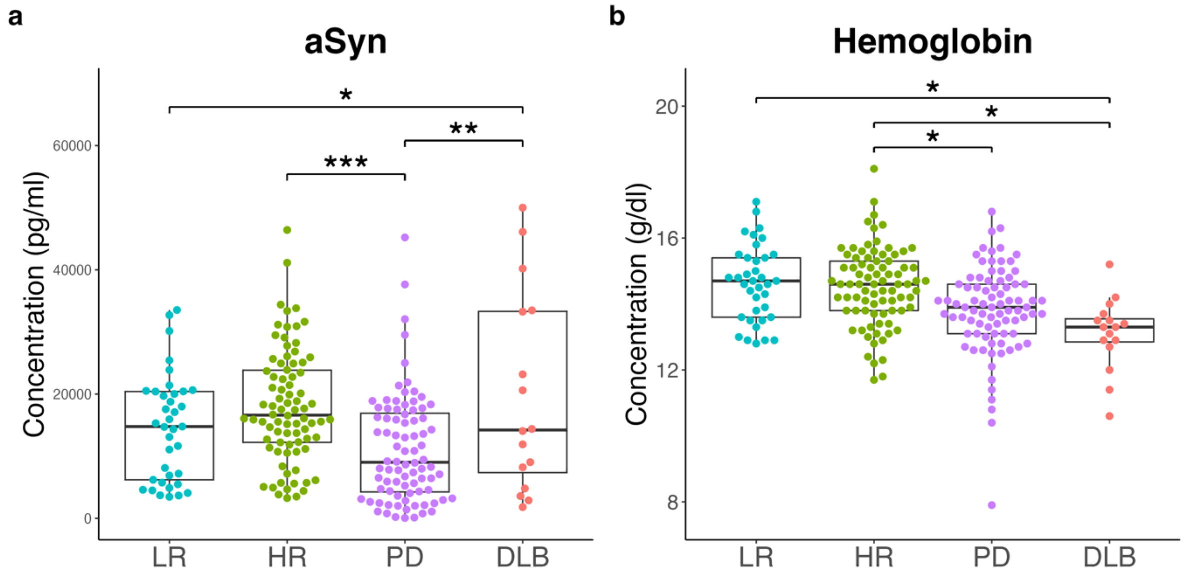

Supplementary Figure 2. Levels of aSyn (a) and hemoglobin (b) are plotted with individual values and boxplots across diagnostic groups. Analysis of covariance (ANCOVA) adjusted for age and sex is used to determine *p* values visualized with ***$p < 0.001$, **$p < 0.01$, *$p < 0.05$. aSyn, alpha-synuclein; LR, low-risk; HR, high-risk; PD, Parkinson's disease; DLB, dementia with Lewy bodies.

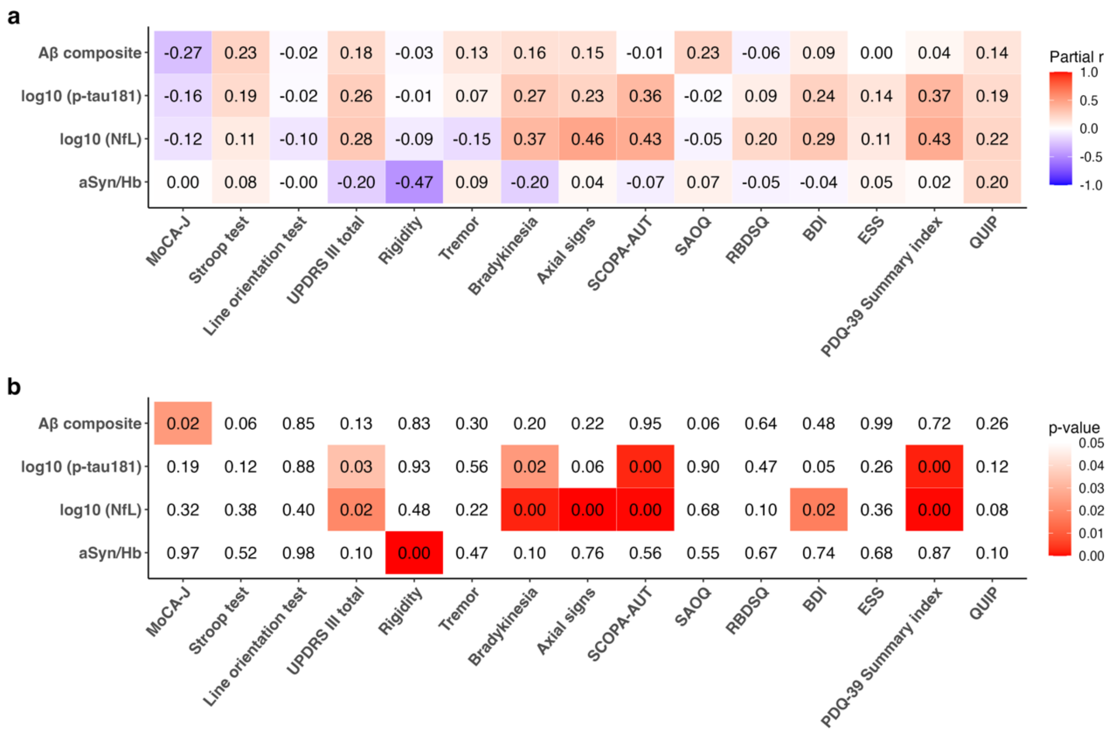

Supplementary Figure 3. Age-adjusted Pearson's partial correlation test between plasma biomarkers and clinical indices in the Parkinson's disease group. Partial correlation coefficients (a) and *p* values (b) are shown. Aβ composite, combination biomarker of amyloid-beta precursor protein $(APP)_{669\text{-}711}$/amyloid-beta $(A\beta)_{1\text{-}42}$ and $A\beta_{1\text{-}40}/A\beta_{1\text{-}42}$ ratios; p-tau181, phosphorylated tau 181; NfL, neurofilament light chain; aSyn/Hb, alpha-synuclein/hemoglobin ratio; MoCA-J, the Japanese version of the Montreal Cognitive Assessment; UPDRS, Movement Disorder Society-Unified Parkinson's Disease Rating Scale; SCOPA-AUT, the Japanese version of the Scale for Outcomes in Parkinson's disease for Autonomic Symptoms; SAOQ, Self-administered Odor Question; RBDSQ, RBD screening scale; BDI-II, Beck Depression Inventory-Second Edition; ESS, Epworth Sleepiness Scale; PDQ-39, Parkinson's Disease Questionnaire-39; QUIP, Questionnaire for Impulsive-Compulsive Disorders in Parkinson's disease

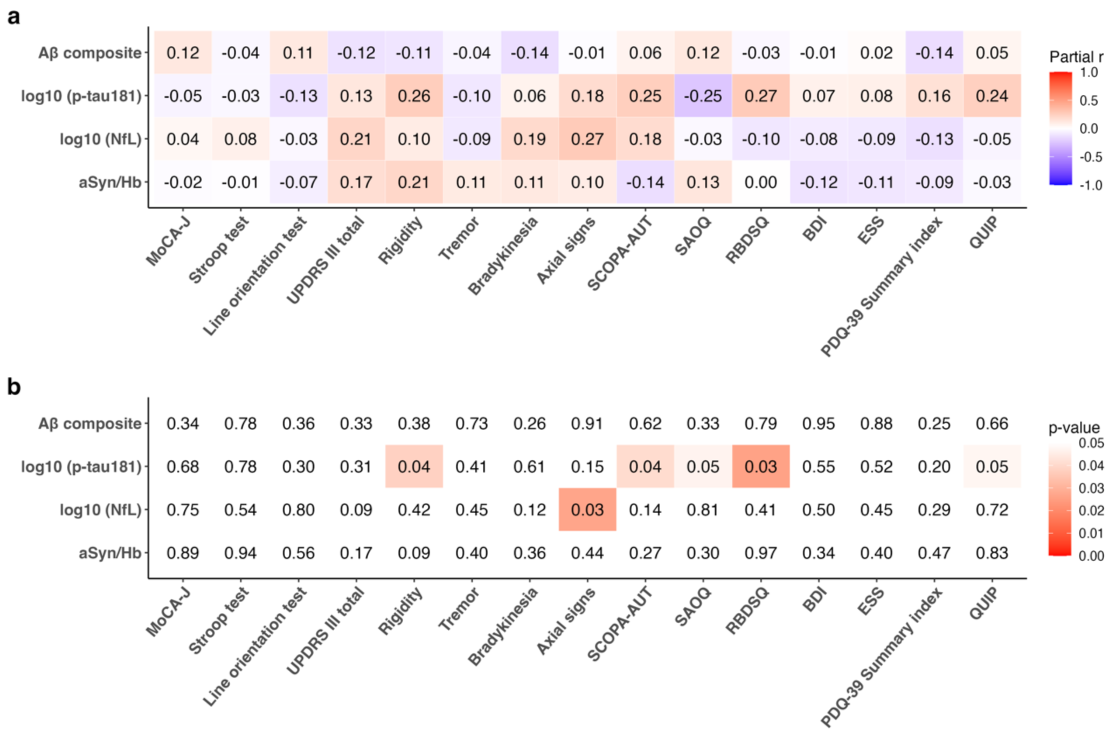

Supplementary Figure 4. Age-adjusted Pearson's partial correlation test between plasma biomarkers and clinical indices in the high-risk group. Partial correlation coefficients (a) and *p* values (b) are shown. Aβ composite, combination biomarker of amyloid-beta precursor protein (APP)$_{669\text{-}711}$/amyloid-beta (Aβ)$_{1\text{-}42}$ and Aβ$_{1\text{-}40}$/Aβ$_{1\text{-}42}$ ratios; p-tau181, phosphorylated tau 181; NfL, neurofilament light chain; aSyn/Hb, alpha-synuclein/hemoglobin ratio; MoCA-J, the Japanese version of the Montreal Cognitive Assessment; UPDRS, Movement Disorder Society-Unified Parkinson's Disease Rating Scale; SCOPA-AUT, the Japanese version of the Scale for Outcomes in Parkinson's disease for Autonomic Symptoms; SAOQ, Self-administered Odor Question; RBDSQ, RBD screening scale; BDI-II, Beck Depression Inventory-Second Edition; ESS, Epworth Sleepiness Scale; PDQ-39, Parkinson's Disease Questionnaire-39; QUIP, Questionnaire for Impulsive-Compulsive Disorders in Parkinson's disease

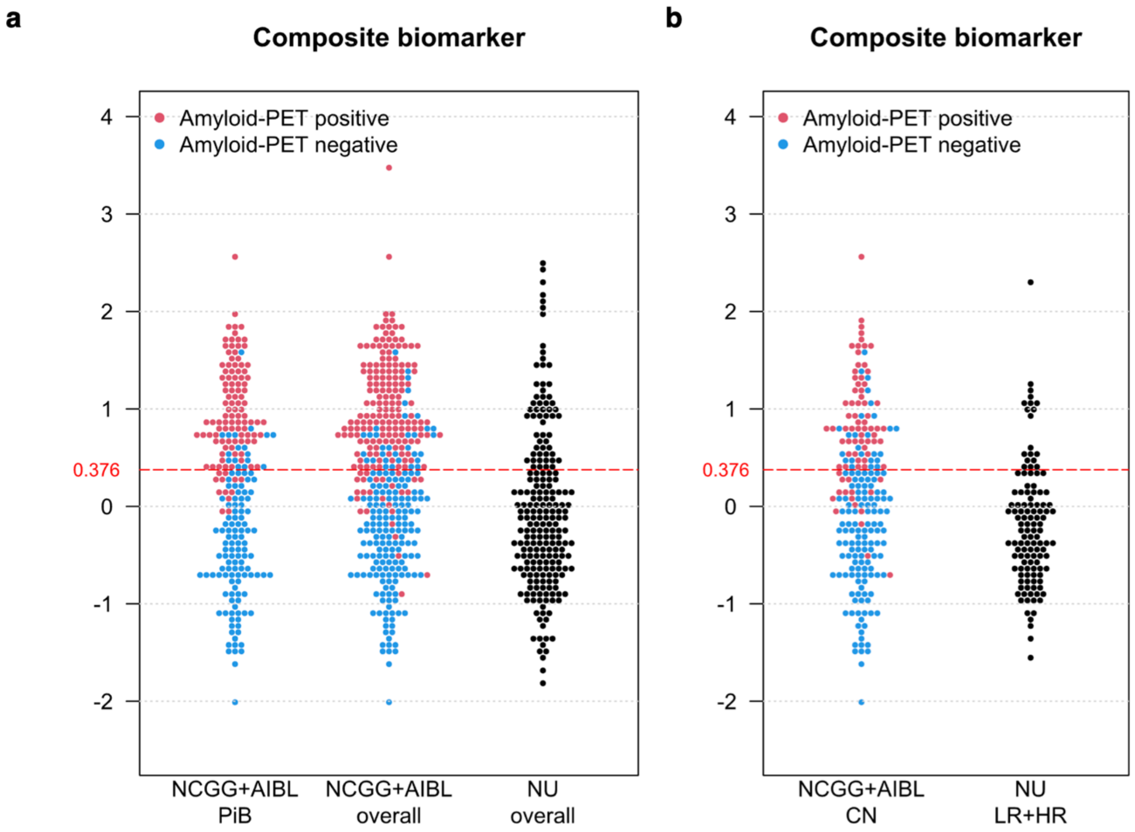

	NCGG+AIBL PiB	NCGG+AIBL overall	NU overall
Number (M:F)	232 (109:123)	373 (182:191)	219 (131:88)
Age, years	74.6 (5.9)	74.1 (5.6)	67.2 (8.7)

NCGG+AIBL CN	NU LR+HR
218 (114:104)	119 (79:40)
73.5 (5.4)	64.5 (6.9)

Supplementary Figure 5. Aβ composite levels in the current cohort (NU) compared to historical cohorts (NCGG and AIBL) in all participants (a) and cognitively normal (CN) participants (b). The cut-off value for the Aβ composite is indicated by the dotted line (0.376). Composite biomarker, combination biomarker of amyloid-beta precursor protein $(APP)_{669-711}$/amyloid-beta $(A\beta)_{1-42}$ and $A\beta_{1-40}/A\beta_{1-42}$ ratios; PiB, Pittsburgh compound-B-amyloid positron emission tomography; NCGG, National Center for Geriatrics and Gerontology; AIBL, the Australian Imaging, Biomarker and Lifestyle Study of Aging; NU, Nagoya University.
Data of age represent the mean (standard deviation).
